# Supplementary material for: ‘A double-edged sword’: Perceived benefits and harms of active case-finding for people with presumptive tuberculosis and communities—A qualitative study based on expert interviews
Source: PLoS One. 2021 Mar 11;16(3):e0247568. doi: 10.1371/journal.pone.0247568 (PMC7951804; doi:10.1371/journal.pone.0247568)
Supplement: Attachment — Submitted filename: Response to Reviewers.docx [file pone.0247568.s004.docx]

**Point-by-point reviewers’ comments and authors’ answers**

Title of the manuscript: “‘A double-edged sword’: Perceived benefits and harms of active case-finding for people with presumptive tuberculosis and communities – a qualitative study based on expert interviews”

**Reviewer comment: 'The methods have previously been described in detail in a study that was based on information from the same interviews' has been cited as ref. no 36, which should be ref no. 37. 'Country income level based on the World Bank’s classification' cited as 36, should be 38.**

Authors’ answer: Thank you very much. We have corrected the referencing as indicated. In addition, we have double-checked all references for their correctness and updated/added three references:

1. WHO. Global TB Report 2020. Geneva: World Health Organization; 2020.

The WHO Global TB Report that we originally included was from 2019. We have now cited the latest Report from 2020. Based on the new Report, we updated the estimated gap between incident and notified TB cases in the world to “2.9 million people in 2020” (instead of 3 million) (p. 4, lines 64-65) and “19% and 83% of TB patients face catastrophic costs” (instead of 27% and 83%) (p. 20, line 422).

2. WHO. Rapid communication on systematic screening for tuberculosis. Geneva: World Health Organization; 2020.

As WHO is in the process of revising their guidelines on systematic screening, the Organization published a rapid communication that was relevant to include. We added the reference (number 18) here: “In this regard, the WHO guidelines for systematic screening contain recommendations for screening specific risk groups for TB [11, 18].”

3. Marks GB, Nguyen NV, Nguyen PTB, Nguyen TA, Nguyen HB, Tran KH, et al. Community-wide Screening for Tuberculosis in a High-Prevalence Setting. N Engl J Med. 2019;381(14):1347-1357.

The trial by Marks et al (2019) is a key reference showing benefits of ACF at the community level. We have now ensured mentioning this reference in the introduction, along another key reference (Fox et al 2018) which we had only mentioned in the discussion section: “At the community level, ACF may lead to reductions in mortality [26], transmission and prevalence of TB [27].” (p. 5, lines 100-101).

Moreover, we have made slight revisions to the discussion section to reference not only the study by Fox et al, but also by Marks et al: “One trial showed an increase of case detection as a result of inviting household contacts to attend a clinic for symptom screening, physical examination and chest radiography, and found reductions in all-cause mortality [26]. The other trial evaluated the effectiveness of community-wide screening compared to passive case-finding and demonstrated reduced prevalence of TB [27].”

**Reviewer comment: Another clarification sought is regarding the questions asked to the participants of this study. Was there any reason for asking about the benefits to the individual and risks at the community level? Both the benefits and risks mentioned in the results pertain to the individual as well as the community level.**

Authors’ answer: Thank you for this observation. We would like to clarify that we did ask about both the benefits and the risks at the individual and community level. We realized that the example questions we had included in the text were misleading. We have therefore rephrased the text as follows: “For example, we asked about the perceived benefits and risks of ACF for the individual, the community, the health system and compared to other interventions for early case detection (S2 File) (instead of: “What are the benefits of ACF in your view, at the level of the individual?”, “What are the risks of ACF from your opinion, at the level of communities?””) (p. 8, lines 168-172)
